# Supplementary material for: Investigation of the Release Mechanism and Mould Resistance of Citral-Loaded Bamboo Strips
Source: Polymers (Basel). 2021 Sep 28;13(19):3314. doi: 10.3390/polym13193314 (PMC8512208; doi:10.3390/polym13193314)
Supplement: Supplementary file 1 [file polymers-13-03314-s001.zip › polymers-1390684-supplementary.pdf]

Supplementary information

Preparation of PNIPAm/Citral nanohydrogels

Briefly, a 250-mL three-neck round bottom flask containing 100 mL of deionised water was purged with high-purity N_2 at room temperature. Then, 0.750 g of NIPAm monomer, 0.038 g of MBA cross-linking agent, 0.038 g of KPS initiator, and 0.038 g of TMEDA catalyst were added. The polymerisation reaction was performed in N_2 atmosphere for 6 h, and, after the reaction, a clear and transparent PNIPAm nanohydrogel solution was obtained following overnight standing incubation. Second, 0.050 g of PEGMS was added to the PNIPAm solution (0.2 mmol/L, below the Critical Micelle Concentration); once it was dissolved, Citral was added for encapsulation reaction. Finally, the solution was immersed in dialysis bags (MWCO: 8000–14000) to remove the unreacted substances and obtain the PNIPAm/Citral nanohydrogel solution, which was stored at room temperature for subsequent use.

Mass of encapsulated citral (L_1)

The Citral mass in PNIPAm/Citral nanohydrogel after dialysis was directly estimated to obtain the Citral mass encapsulated in the PNIPAm nanohydrogels. Briefly, 20 mL of anhydrous ethanol was accurately measured in a 50 mL centrifuge tube, to which 0.5 mL of PNIPAm/Citral nanohydrogel solution was added, and the mixture was centrifuged at 20°C for 10 min at 15,000 rotation/min. Immediately after centrifugation, the supernatant was collected to determine the absorbance of Citral (λ_{max}) on the UV–Vis spectrophotometer. Finally, the mass of encapsulated Citral (L_1) was calculated from the standard curve of Citral in anhydrous ethanol solution.

Table S1. Classification Standard of Surface Infection Levels of Samples

**Levels**	**Infected Area of Sample**
0	No hyphae or mildew on the sample surface
1	Infected area of sample < 1/4
2	Infected area of sample 1/4–1/2
3	Infected area of sample 1/2–3/4
4	Infected area of sample > 3/4

Table S2. Factor level

**Level**	**Factor**		
	A (mg)	B (h)	C (°C)
1	10	1	25
2	30	2	30
3	50	3	35
